# Thematically weighted regression models for identification of important drivers of environmental trends in lake survey data

**DOI:** 10.1007/s10661-025-14611-4

**Published:** 2025-10-16

**Authors:** Claudia von Brömssen, Jens Fölster, Karin Eklöf

**Affiliations:** 1 Division of Applied Statistics and Mathematics, Department of Energy and Technology, Swedish University of Agricultural Sciences, PO Box 7032, 750 07 Uppsala, Sweden; 2 Section for Geochemistry and Hydrology, Department of Aquatic Sciences and Assessment, Swedish University of Agricultural Sciences, PO Box 7050, 750 07 Uppsala, Sweden

**Keywords:** Environmental change, Grouping of stations, PH, Low temporal monitoring frequency, PCA, PLS

## Abstract

The processes that drive environmental change are complex and often interwoven. Monitoring such processes and their effects on environmental variables is, at best, spatially and temporally incomplete. The Swedish Lake survey consists of several thousands of randomly selected lakes stretching over a wide range of potential influences, including regional and local scale pressures, such as recovery from acidification, changes in climate, and effects from various catchment characteristics. The main drawback for this monitoring data is that the temporal resolution is low and does not allow for the use of traditional trend evaluation methods at individual stations, much less the attribution of important drivers. In this study, we present a method that enables an evaluation of important drivers of change by defining trend coefficients as smoothed estimates over lakes that exhibit similar attributes, e.g., comparable levels or similar temporal changes in selected explanatory variables. The principles are the same as geographically weighted regression but replace the geographic coordinate system with a thematic one, based on principal (PCA) or partial least squares (PLS) components. We illustrate this method by evaluating trends in pH in Sweden from 2012 to 2023 and detected several regions where pH is decreasing, mainly in relation to changes in calcium.

**Supplementary Information:**

The online version contains supplementary material available at 10.1007/s10661-025-14611-4.

## Introduction

Identifying the most relevant drivers for environmental change is crucial to determine sufficient countermeasures, but it is a complex task when there are numerous potential causes ranging throughout the temporal and spatial scale which can amplify or weaken each other. How well a connection between potential drivers and the level of temporal change of an environmental variable can be established is dependent on several factors, not least the temporal and spatial frequency of measurements in relation to the complexity of underlying processes. Temporal trends are often studied in time series with seasonal, or in some cases more frequent, observations. However, such stations are often too limited in number to cover a wider range of influences of potential drivers. On the other hand, in surveys including a large number of lakes the temporal sampling frequency is usually too low to evaluate trends for single sites. von Brömssen et al. (2023a) suggested the use of geographically weighted regression (GWR) models to accomplish spatially smoothed trends when temporal data is sparse. In this study, we will extend this work to investigate the effect of drivers aside from geography.

Attribution of influences by potential drivers on trends in time series with reasonable temporal frequency, i.e., monthly or biweekly measurements, is typically studied within individual series. This can be carried out by simply visualizing trend curve estimates side-by-side for the variable of interest and the drivers (Erlandsson et al., 2008; Monteith et al., 2014; von Brömssen et al., 2021). Alternatively, regression models can be used to describe effects of riverine flow on concentrations of various compounds. The slope of this concentration–discharge (C-Q) relationship is then used to identify dominant sources and delivery pathways for solutes and particulates. Statistical models range from simple linear regression (Godsey et al., 2009), piecewise linear regression (Muggeo, 2003; Rose et al., 2018), to nonlinear regression and time-varying relationships (Dehaspe et al., 2021; von Brömssen et al., 2023b). When several explanatory variables are simultaneously accounted for multiple linear regression is a commonly used method (Asmala et al., 2019; Erlandsson et al., 2008).

To investigate the spatial structure of potential drivers, i.e., to distinguish which lakes or rivers reveal specific patterns in temporal change, the analysis is often conducted stepwise. First, a summary measure of temporal trend is computed for each station, then stations with similar summaries are grouped and characteristics of these groups of waters are identified. The most commonly studied summaries of temporal change are (i) absolute or relative changes over a predetermined period of time and (ii) estimated trend components, such as Theil-Sen slopes or regression slope coefficients. Indeed, Asmala et al. (2019) used relative change in total organic carbon (TOC) from 1993 to 2017 for 30 rivers and connected them to land use proportions in a linear regression. Lepistö et al. (2021) used percentage and absolute change in TOC as a response variable and related them to temperature, absolute change in sulfate, and percentage of forest drainage. Trend summaries were also used by Räike et al. (2024) who extracted a 0/1-variable indicating “no trend” and “upward trend” from 746 monitoring stations and used random forest algorithms to connect this variable to drivers representing hydrology, meteorology, land use, and forest cover.

For such approaches, either the absolute level or the absolute or relative temporal change of potential drivers also must be summarized per site. They may represent the same time period that was selected for the identified trend, but other periods can also be used. For example, Erlandsson et al. (2008) used moving averages of antecedent stream flow and air temperature of different length as an explanatory variable in a linear regression for concentration levels of organic matter in 28 rivers and compared fits and coefficients across catchments. Imtiazy et al. (2020) used summarized explanatory variables, such as sulfur deposition and precipitation, at different levels (annual or seasonal with and without time lag) to explain changes in dissolved organic carbon (DOC) levels. Moreover, the average of the response variable during a preceding time period can be a relevant explanatory variable, particularly if the trend represents a recovery from earlier pressure or if certain areas have been more exposed for a longer duration. For example, Futter et al. (2024) and Huser et al. (2018) found that negative trends in total phosphorus concentrations were stronger in already phosphorus-poor rivers and lakes in Sweden. Summaries on different spatial scales for drivers can be equally important (Tzanopoulos et al., 2013). Therefore, Imtiazy et al. (2020) distinguished between local and regional impacts by selecting local variables, such as on-site precipitation and deposition, and regional variables, consisting of different oscillation indices, when modelling DOC concentrations in 49 eastern Canadian lakes.

When the number of potential drivers is high or when several related temporal or spatial summaries are used, the selected explanatory variables can be highly correlated with each other. Principal component analysis (PCA) can then be used to estimate factors from correlated explanatory variables, which in turn can be used in a traditional regression model (PCAR). Partial least squares (PLS) works similarly but takes the response variable directly into account when constructing the factors. Also, stepwise variable selection methods have then been used. Abel et al. (2021) used PCA to identify the relative importance of climatic and anthropogenic variables on changes in vegetation sensitivity. Ryu et al. (2021) identified external forcing agents of environmental change using PCA on concentration of, among others, Cl, Ca, and Fe, and several taxonomic groups. Moreover, de Wit et al. (2023) used PCA to study the covariation in changes in land cover and chemical components, and PLS to determine which changes in chemistry could be best explained by land cover and changes in climatology and chemical variables. Evans et al. (2005) modelled TOC levels using rainfall, temperature, pH, SO_4_, Cl, and the sum of acid anion concentrations (SO_4_ + Cl + NO_3_) for 11 lakes and 11 water courses using a stepwise multiple linear regression model.

Models based on temporal summaries for site-specific characteristics can be constructed even if the temporal resolution of data is low. In this study, we investigated to which extent we can connect temporal change in pH to relevant drivers in a data-driven approach based on sparse temporal data. We used measurements from the Swedish Lake Survey (SLS), a monitoring program covering 6230 randomly selected lakes with a revisit interval of 6 years and a monitoring time span from 2007 to 2023 (Fölster et al., 2014b). Temporal trends in a spatial perspective for this dataset have been previously studied (von Brömssen et al., 2023a) using geographically weighted regression models (Brunsdon et al., 1998). However, using only the geographical location as indicator for change might be naïve since lakes in Sweden show high local diversity caused by catchment size, lake surroundings, and variations in soil that can directly influence lake chemistry. In the present study, we suggest a methodology to connect temporal trends to gradients defined by potential drivers rather than geography. We first apply a dimension reduction technique, like PCA or PLS, and use the obtained factors as basis for the weighted regression that estimates the temporal trend.

The method is illustrated by investigating trends in pH from 2012 to 2023. Increasing pH-trends for time series starting in the 1990 s or earlier have been broadly observed as a reaction to recovery from acidification (Futter et al., 2014; Garmo et al., 2014; Minella et al., 2013; Monteith et al., 2014; Vuorenmaa et al., 2018). In the past decade, however, both the magnitude and direction of trends that were observed varied substantially throughout Sweden (von Brömssen et al., 2021). Thus, a more thorough analysis of contemporary trends in pH would be useful, since pH is important for biodiversity in the boreal region and might be influenced by many different natural processes and anthropogenic activities (Grennfelt et al., 2020). Temporal trends in pH are hypothesized to be driven by reduced levels of sulfur deposition (Garmo et al., 2014; Vuorenmaa et al., 2018) as well as by changes in climate and increased eutrophication (Minella et al., 2013). Levels of pH in Sweden are generally low due to the high levels of natural organic acids and can vary due to stream flow conditions (Erlandsson et al., 2011). Observed station-wise data from the SLS was evaluated in a weighted regression context, where the following variables were included as potential drivers:

- The initial state of the response variable pH
- The initial state of the selected environmental variables describing, e.g., the recovery from acidification and changes in climate
- The simultaneous temporal change in the same environmental variables
- General information regarding land use in the catchment, as well as catchment size

This study tests whether the proposed methods can reliably link multiple, potentially correlated explanatory variables to prevailing trends and evaluates if any approach is preferable, using the complexity of temporal pH trends as case study.

## Material and methods

### Weighted regression models

Weighted regression is an extension of traditional least squares regression that allows different observations to be assigned different weights, i.e., increasing or decreasing their importance in the model fit. This concept is used in geographically weighted regression to weight observations within a geographical window, with respect to the distance from the window’s center. In the following sections, we describe the basics of geographically weighted regression models and suggest how geographical coordinate systems can be replaced by coordinates that represent different combinations of potential explanatory variables, which in turn will be used to study temporal trends. This approach will be termed thematically weighted regression (TWR) models.

#### GWR models 

Geographically weighted regression models are used to model a response variable as a function of one or several explanatory variables ${x}_{1}, {x}_{2}, \dots , {x}_{p}$ (Brunsdon et al., 1998):

$${Y}_{l}={\beta }_{l0}+\sum_{k=1}^{p}{\beta }_{lk}{x}_{lk}+{\varepsilon }_{l}$$

${\beta }_{lk}$ are the regression coefficients for the variable ${x}_{k}$, where $l$ indicates the spatial location. The error term in the model is assumed to follow a normal distribution with mean zero.

To determine where in a geographical area a variable is changing over time, von Brömssen et al. (2023a) adapted the model by using time as the main explanatory variable. The model is then

$${Y}_{l}={\beta }_{l0}+{\beta }_{l}\bullet t+{\varepsilon }_{l}$$

where $l$ again denotes the geographical location and ${t}$ is a time variable, typically the year of observation. ${\beta }_{l}$ represents the regression coefficient, which represents the change in the response per time unit and is dependent on location.

In practice, for each spatial location a spatial window or neighborhood is defined. Within each window, a linear regression model is fitted, including weights that describe the distance of the individual data points to the center of the window, which is the target location. The estimated regression coefficient represents this location. Using weights allow for a smooth change in regression coefficients over space as observations on the edge of the spatial window have low weights. Due to the typically uneven sampling of environmental data, the neighborhood is often chosen to consist of the *k* nearest neighbors rather than fixed distances from the target location. A typical choice for the weighting function for the included observations is bi-square weighting:

$$w_{lm}=\left\{\begin{array}{cc}\left(1-\left(d_{lm}/b\right)^2\right)^2&if\;\left|d_{lm}<b\right|\\0&otherwise\end{array}\right.$$

where ${d}_{lm}$ denotes the distance between observation *m* and the target location $l$, while *b* represents the neighborhood size. Typically, the distance measures in geographical weighting are selected to be Euclidian.

Geographically weighted regression models will be used to show geographically differentiated temporal trends for pH following the principles described by von Brömssen et al. (2023a).

#### TWR models

When processes other than geographical location are expected to drive environmental change, the geographical coordinate system in a GWR could be replaced by a coordinate system that is defined by such underlying driving processes. For this, we can use dimension reduction techniques, such as PCA or PLS, on a dataset containing station-wise summaries of variables that describe recovery from acidification, changes in climate, or catchment properties. The coordinates, i.e., scores, from such a model can replace geographical coordinates in a weighted regression context. This means that the k nearest neighbors of a lake would be identified as lakes that are similar in their level or change in chemistry, climate, and/or catchment characteristics and the weights in the weighted regression model are determined by distances in the same coordinate system. Thus, the goal of the analysis is to investigate if temporal trends are notably prominent in selected areas defined by these thematic coordinates.

##### Using PCA to define a coordinate system

PCA (e.g., Johnson & Wichern, 2019) identifies hyperplanes in the multidimensional space that approximate the data and maximize the explained variance of the variables included. PCA is unsupervised, meaning that no response variable is included. The components are orthogonal to each other and can be directly used as a coordinate system with the value of the observations in the coordinate system given as the PCA scores. Which of the original explanatory variables are predominantly represented by the different principal components can be studied using the PCA loadings. The variables included and pre-processing of these variables is described in the “Data” section, “Data pre-processing” section, and Table S.1. Since the variables included have different scales, they are standardized to mean 0 and variance 1 before the PCA is conducted.

##### Using PLS components as a coordinate system

Similarly, PLS (Wold et al., 2001) identifies components that reduce the dimensions of the original dataset. By using PLS, the components are extracted to maximize the covariances between the explanatory variables and one or more response variables. It is therefore necessary to define a goal variable, such as a site-specific quantification of the prevailing trend, to define a relevant coordinate system. For this, an average annual change for pH was computed as the difference between the last and the first observation in time divided by the number of years between these observations (“Producing station-specific covariates” section). The explanatory variables were the same as for the PCA and are standardized to mean 0 and variance 1 prior to the analysis.

#### R packages

The fitting of weighted regression models, both on the geographical and on the thematic coordinate system, was carried out using the readily available software package GWmodel (Gollini et al., 2015; Lu et al., 2014) in the free statistical software R (R Core Team, 2025). The size of the k-nearest-neighborhood (knn) was determined by leave-one-out cross-validation and a bi-square kernel was selected as the weighting function. The PCA was performed using the function prcomp, while additional outcome and visualizations were constructed using the package factoextra (Kassambara & Mundt, 2020). PLS was fitted using the package plsdepot (Sanchez, 2023).

The R scripts and data used in this study are available on GitHub (https://github.com/claudiavonbromssen/TWR-for-identification-of-drivers).

### Data

**Fig. 1:**
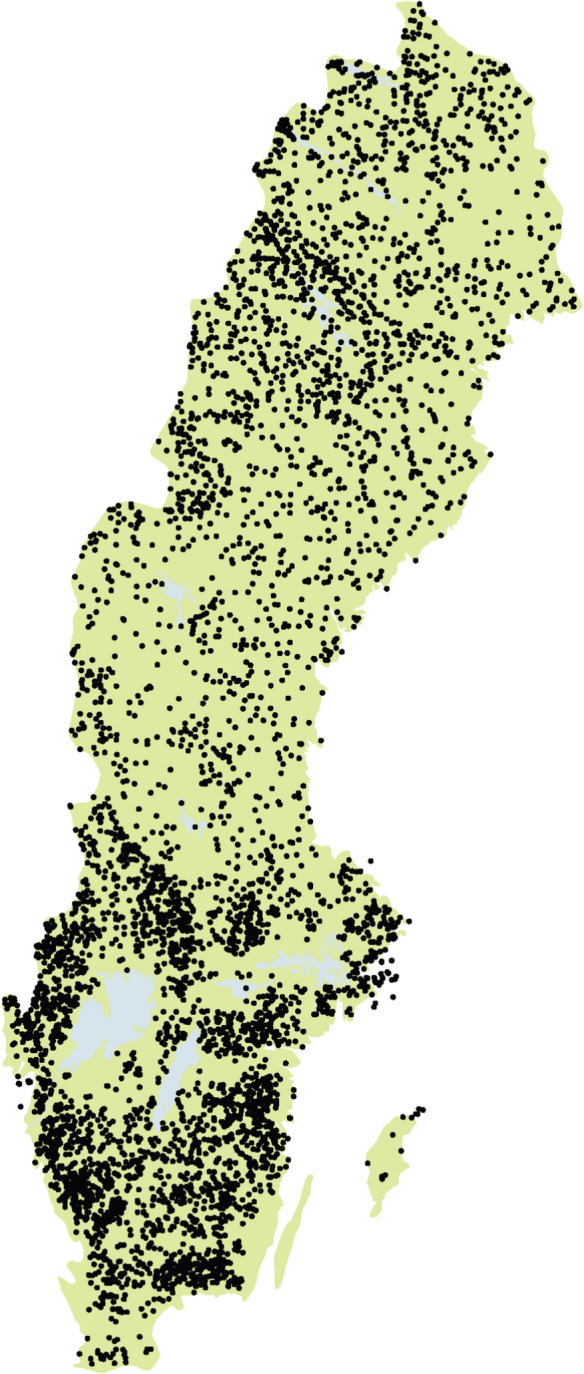
Geographical distribution of the lakes monitored in the Swedish Lake Survey

The national monitoring program the SLS includes approximately 4800 lakes larger than 0.01 km^2^ from the national lake register within geographical and lake size strata (Fölster et al., 2014a, 2014b). Additional lakes were selected regionally by the same principles at different extents throughout the years, resulting in a total of about 6230 lakes. The lakes cover a gradient in climate and impact with higher temperature and stronger impact from air pollution, land use, population, and industries in the south compared to the north. To account for the higher diversity in anthropogenic impact, the geographical stratification of the lakes allowed a larger number of lakes in southern Sweden (Fig. 1).

The programs have a revisit time of 6 years, meaning that a single station has one observation every 6 years and each year about one sixth of the lakes are monitored. Mid-lake surface samples are taken during autumn circulation, which usually occurs between September and December, since these measurements are considered to be representative of the entire lake (Göransson et al., 2004). Occasional late observations (up to January) are attributed to the autumn of the previous year. During the autumn of 2018 and January 2019 fewer lakes were sampled due to the technical breakdown of the helicopter that is used for their monitoring. The remaining lakes were sampled together with the panel planned for 2019. Certain data quality problems were detected for the first year of monitoring in the SLS (2007); therefore, only data from 2008 was included in this study.

In the present study, pH, alkalinity, conductivity, magnesium (Mg), chloride (Cl), calcium (Ca), sodium (Na), and sulfate (SO_4_) were selected to describe a lake’s chemistry. pH is selected as the variable of interest to be modelled, while the remaining variables are used as explanatory variables. Alkalinity describes the buffer capacity to resist acidity and is determined by titration to a fixed endpoint of 5.6 (SS-EN ISO 9963–2:1994), while Ca and Mg represent the contribution of soil weathering to alkalinity. Conductivity indicates the total ionic concentration. SO_4_ reflects the impact from acid deposition and Cl the impact from marine influences, respectively. Na may origin from both soil weathering and from marine influence.

Additionally, information concerning the prevalent land use in the different catchments was available from the national land use map (Naturvårdsverket, 2020) aggregated into 15 classes following the national reporting to HELCOM (Widén Nilsson et al., forthc). These variables describe the composition and management of forests, as well as the amount of urban and arable land in the area, which can affect levels of lake pH. Concentrations or organic acids could be naturally high in catchments with a high share of peatlands. Land-use activities such as forest harvest could also influence pH values due to changes in flow pathways that mobilize organic acids from soil to surface waters (Schelker et al., 2012). Air temperature and precipitation data were downloaded as annual mean values between 1990 and 2020 from the Swedish Meteorological and Hydrological Institute (SMHI Open Data PTHBV; SMHI, n.d.) and were included to describe effects of spatial differences in climate and effects of climate change.

### Data pre-processing

Data between 2012 and 2023 was selected for the trend analysis. This period is short enough to make a reasonable assumption of an approximately linear trend, while we still have at least two observations for each station. The earlier years of the survey (2008–2013) are used to define initial levels of both the response and explanatory variables. Basic pre-processing of the data and the construction of relevant summaries is described in the following sections. In total, 36 explanatory variables were constructed, 8 of which represented change, 10 initial levels, and 18 constants (Table S.1).

#### Basic pre-processing

Climatic data and catchment characteristics were available for 5127 lakes. Stations that had less than two observations throughout 2012 to 2023 were removed to ensure that only time series type of data was included when trends were computed. Additionally, two lakes (Vitvattentjärnen and Stensjön) were removed due to unreasonable values in observed levels or changes. This resulted in a final number of 4962 lakes for further analysis.

All water chemistry variables except pH were log_10_-transformed prior to analysis to account for the skewed data distribution and to decrease the effect of potential outliers. Spatial variables that represented the percentage of land use were transformed by a centered log ratio transform (Filzmoser et al., 2010) to enable their representation in the Euclidean space. This was conducted using the package compositions in R (Van den Boogaart & Tolosana-Delgado, 2008).

Values below the reporting limit were replaced with half of the reporting limit’s value (USEPA, 2000). For Cl, where the reporting limit changed over time, all values at or below the highest reporting limit were replaced with half its value.

#### Producing station-specific covariates

The goal of defining a new coordinate system is to establish where individual lakes are placed in relation to their water chemical, climatic, and landscape properties. We distinguished between three general types of identified site-specific covariates for the trend analysis: (i) constant values, (ii) values that define the initial level of an otherwise varying covariate, and (iii) values that define the temporal change of covariates.

##### Constant lake-specific covariates

The constant covariates that were used were the size of the catchment and elevation. Percentages of land use within the catchments, such as the percentage of arable land or forested areas (Table S.1), were only available once for each lake and are therefore also considered constant.

##### Initial level of lake-specific covariates

Initial levels of chemical variables were computed for the period 2008 to 2013 to evaluate if stations with particularly high or low initial levels in certain variables tend to show a stronger change in the response variable. For several stations, no observations were available for this period, and therefore, the first value after that was used if it was observed before 2018. Similarly, the initial level of pH is determined for each station and added as explanatory variable.

For both temperature and precipitation, annual averages were available between 1980 and 2020. We computed initial levels as mean values for the same period that was used for the chemistry variables (2008–2013). Further, we computed an overall mean for all years. Although the 6-year mean of initial levels and the overall mean were expected to be similar, using separate summaries provided us with the possibility to distinguish effects if the period between 2008 and 2013 deviates from the long-term mean.

##### Covariates describing lake-specific change

To quantify temporal changes in the available chemical variables from the SLS, excluding pH and alkalinity, we computed the average annual change as

$${Variable}_{change}=\frac{{y}_{j}-{y}_{i}}{j-i},$$

where *y* indicates the variable in question and *i* and *j* are the years where observations were made. For this, the first and last observations during 2012 and 2023 were used.

For the variables temperature and precipitation that are measured with a higher time resolution, the Theil-Sen slope was computed for the period 2012–2020, because data after 2020 was not available.

#### Preprocessing of response variable

The response variable in TWR consists of the individual measurements of pH over time per station between 2012 and 2023. To reduce the variation between pH levels of individual lakes the data is station-wise mean centered, as described by von Brömssen et al. (2023a) before analysis.

## Results

### Thematically weighted regression models on a PCA coordinate system

**Fig. 2:**
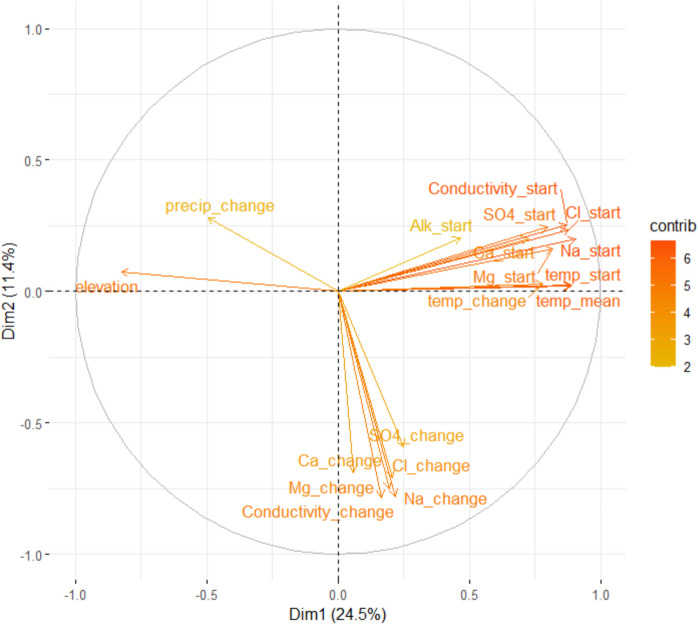
The first two principal components defined for the explanatory variables by PCA. The color of the arrows indicates the extent to which they contribute to the two components, with red colors and long arrows indicating strong contributions and short and blue arrows indicating either no or little contribution. Only the 18 most important variables are shown for readability

Principal components were extracted and visualized pairwise in components plots using variables describing initial levels and change in chemical and climatic variables, as well as constant catchment properties (Table S.1). The first four components explained 24.5%, 11.4%, 9.7%, and 8.2% of the variation in the included variables, respectively. The first component was tightly connected to initial values of many of the included chemical variables, together with elevation and average temperature, indicating a north–south gradient. The second component co-varied with variables indicating temporal change in chemical variables that describe ionic strength (Fig. 2; Figure S.1, Table S.2). The third component had a connection to the initial levels of pH and alkalinity and the percentage of deciduous forests and was inversely related to percentages of other types of forests and forests on wetlands (Figure S.2). The fourth component was connected to the overall level of precipitation and the percentage of artificial surfaces.

**Fig. 3:**
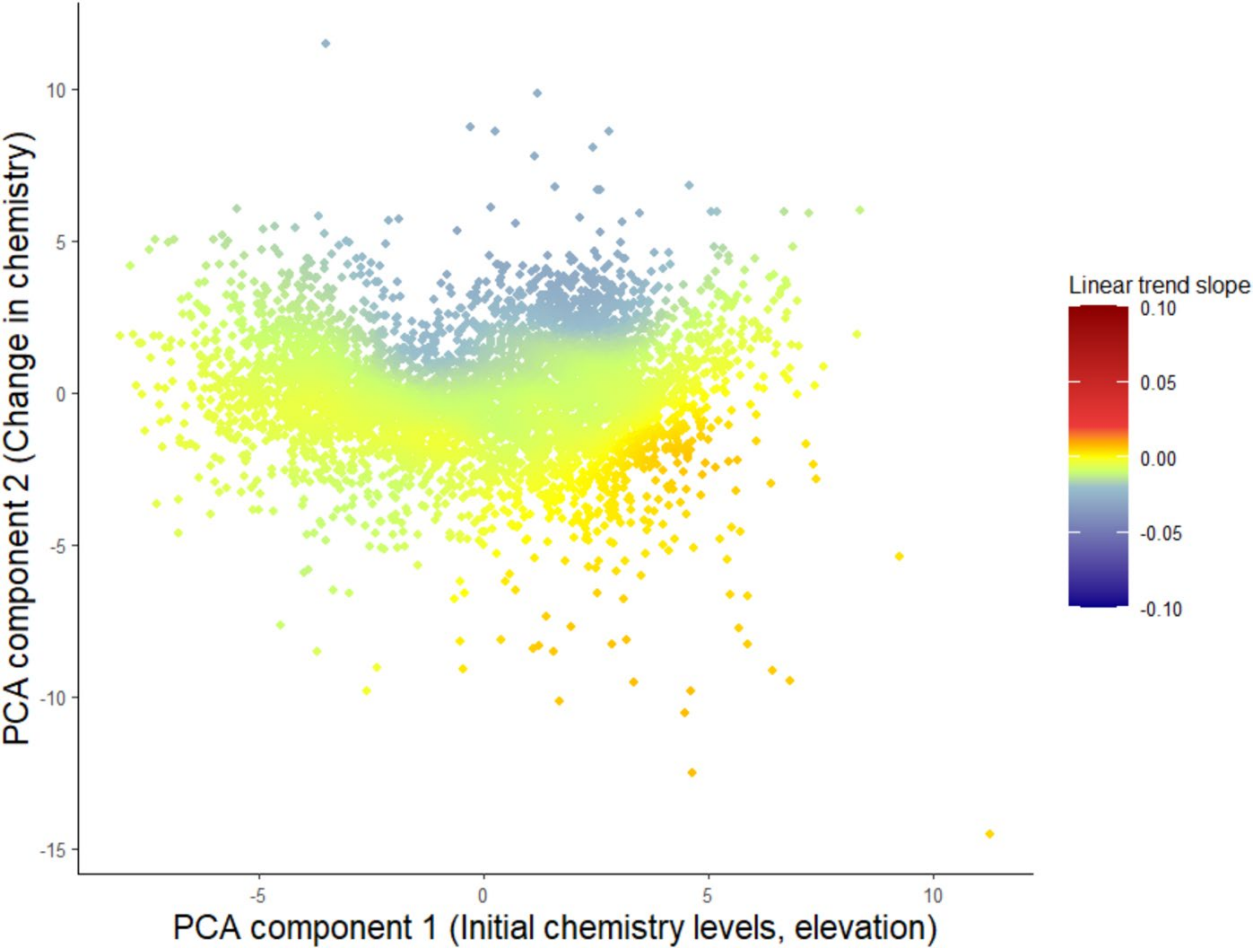
The results of the TWR for trends in pH 2012–2023 on the first two PCA coordinates. Using cross-validation a bandwidth of 1224 (knn) was selected. Positive trends are given in red and negative trends in blue. The *x*-axis represents the first principal component and the *y*-axis the second principal component equivalent to the components presented in Fig. 1

A TWR was computed on thematical windows based on the PCA results. The first two PCA-components were selected, and observation scores were extracted and used as coordinates in the weighted regression, where station-wise centered values of pH for the years 2012–2023 were used as the response. The size of the windows, i.e., how many observations were analyzed together, was determined by cross-validation. The analysis shows that negative trends in pH were strongest when the second PCA-coordinate was positive (Fig. 3, blue). These stations had negative changes in Cl, Ca, Na, Mg, conductivity, and SO_4_. They were more strongly negative when the first PCA-coordinate did not have very high or very low values, i.e., if initial values of variables of ionic strength and temperature, as well as elevation, was medium, which could be translated to locations in Mid to Southern Sweden. The fitted TWR showed local *R*^2^ values of about 0.28 in this area and estimated trend slopes were approximately − 0.03 units per year. Positive trends in pH were both generally weak and observed when the initial values of variables describing ionic strength were high and when changes in these variables were positive.

PCA components were optimized without accounting for a response variable. Therefore, we also fitted TWR using scores from PCA components 3 and 4 to determine if any of the later components carry important information about pH trends. These components identified trends with magnitude of at most − 0.02 units per year, indicating a gradient in the third component, with the highest negative trends where initial levels of pH and alkalinity were low (Figure S.2). The highest local *R*^2^ values in this model were approximately 0.13.

### Thematically weighted regression models on a PLS coordinate system

**Fig. 4:**
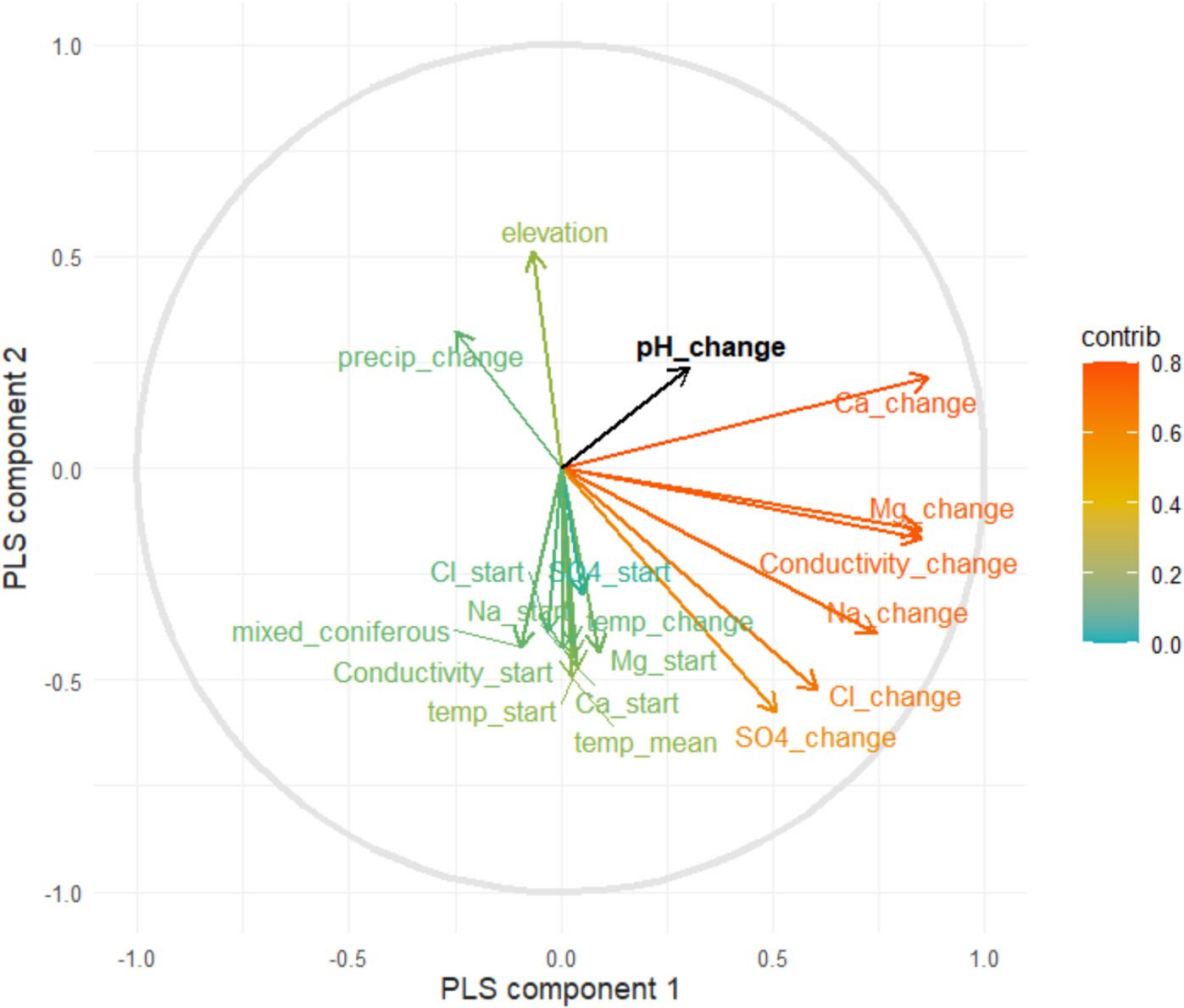
The first two components defined in the PLS. The color and length of the arrows indicate the extent to which different variables contribute to explaining the variation in the two components, using *R*^2^ values as an index. Only the 18 most important variables are shown for readability

Similarly, the TWR can be fitted on a coordinate system that is based on PLS components. The PLS approach used change in pH as a response variable and determined the components that maximized covariation between this change and the explanatory variables. Again, scores were extracted for the first and second component and used in a TWR model, where the observed station-wise centered values of pH were used as response variable. The first PLS component was primarily determined by changes in chemical variables notably Ca, Mg, and conductivity. The second component is connected to elevation and temperature, which represent both a north–south gradient and was also connected to the initial values of Ca, Cl, Mg, Na, SO_4_, and conductivity (Fig. 4; Figure S.4, Table S.3). Changes in SO_4_ and Cl were more closely related to the second component than to the first. The first two components explain 9.1 and 5.5% of the variation in pH, respectively. It is important to note that this is not comparable to the explained percentages by PCA, which are in regard to the variation in the explanatory variables, and not in a response variable.

**Fig. 5:**
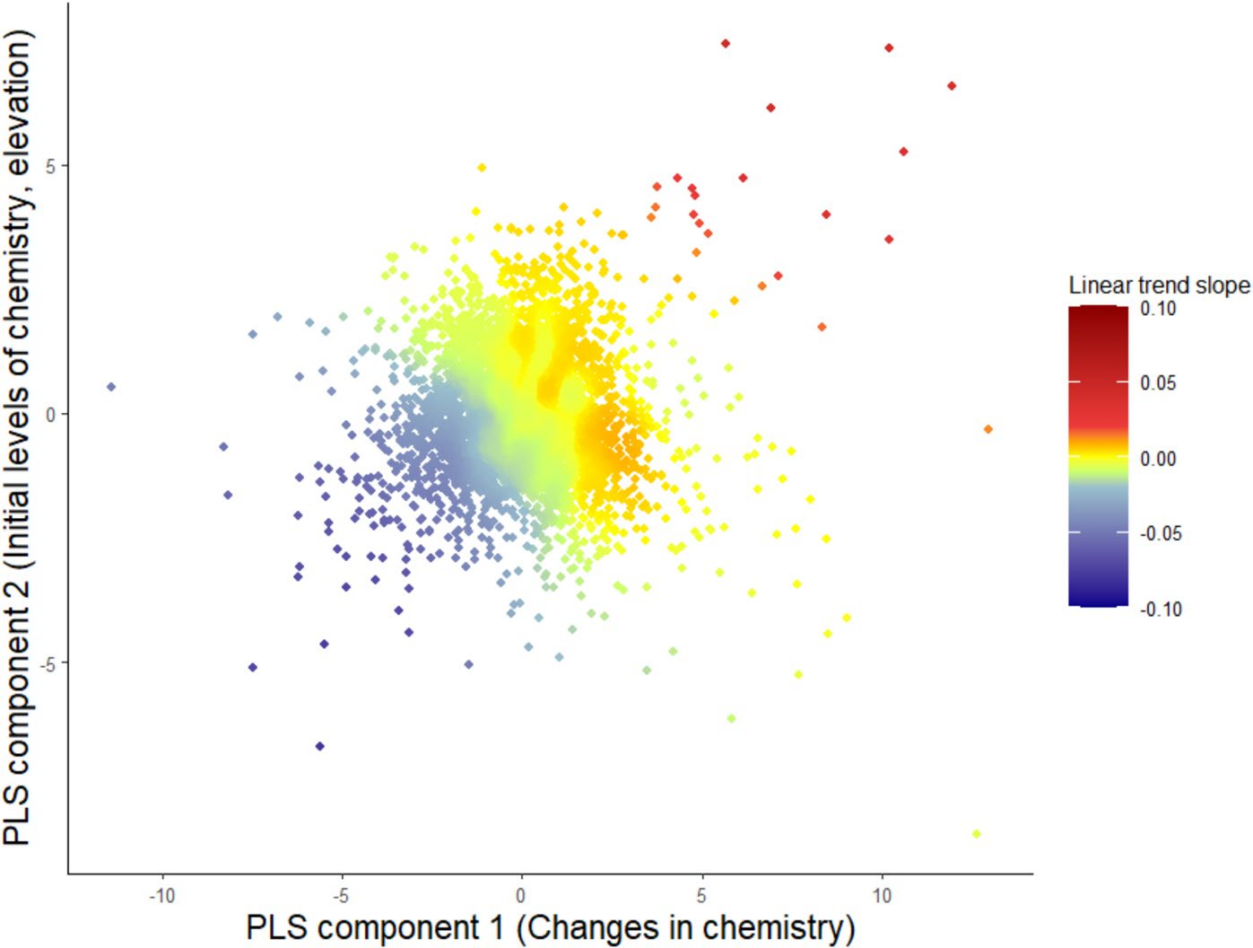
The results of the TWR for trends in pH 2012–2023 on the first two PLS components. Using cross-validation a bandwidth of 602 (knn) was selected. Positive trends are shown in red and negative trends in blue. The *x*-axis represents the first PLS component and the *y*-axis the second PLS component equivalent to the components presented in Fig. 4

The trend estimates in the PLS-based TWR showed stronger negative trends compared to the PCA-based analysis. The strongest identified trends indicated a change as large as − 0.075 units per year and positive trends of approximately 0.036 units per year (Fig. 5). Negative trends in pH were observed when changes in Ca, Mg, and conductivity were negative, and the percentage of deciduous forest was small. Changes in SO_4_ and Cl were predominantly connected to the second PLS component, i.e., positive changes in SO_4_ and Cl were connected to negative trends in pH, but this connection was not strong. Local *R*^2^ values were as high as 56% where strong negative pH trends were observed.

Certain stations lay further away from the bulk of observation, which could influence how PLS components were determined, and which trends were identified in the TWR (Fig. 4). However, an analysis after omitting the 18 most deviating observations did not lead to any important changes in the overall results. In this analysis, several of the stations that exhibited the highest positive changes were removed and the remaining stations showed smaller increases at a maximum of 0.013 units per year (not shown). Conclusions for areas with negative trends were not affected.

### Comparisons of thematic-based and geography-based temporal trends

**Fig. 6:**
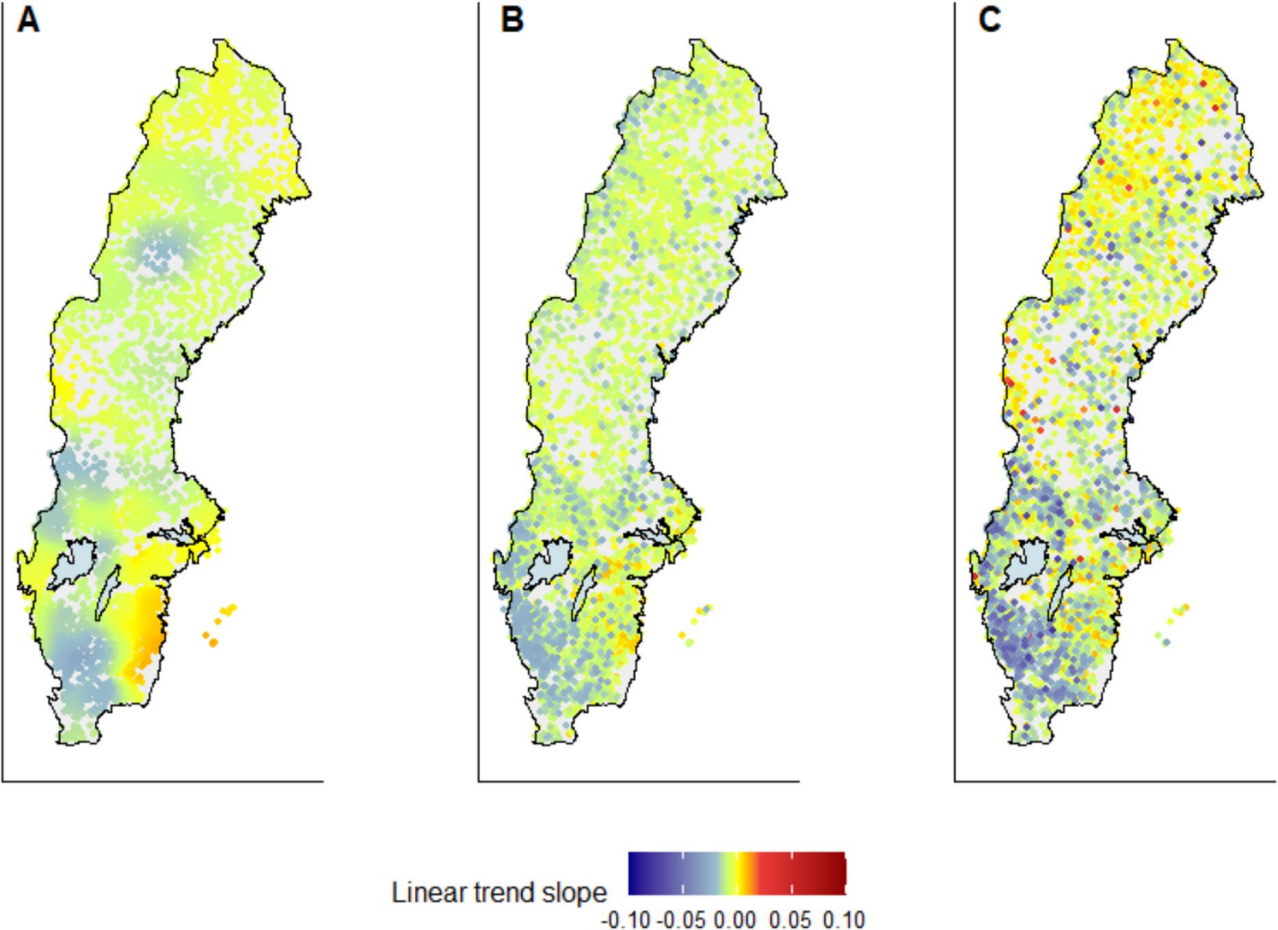
Left (**A**): Geographically weighted pH trends in Sweden (2012–2023). Mid (**B**) and right (**C**): Thematically weighted pH trends in Sweden (2012–2023) based on PCA coordinates and PLS coordinates, respectively, presented in a geographical perspective

Estimated trend slopes obtained from a TWR can be presented on geographical coordinates to better understand spatial patterns of both response and explanatory variables. For comparison, we conducted an analysis using only geographical coordinates as drivers, i.e., we used a standard GWR model to identify spatially differentiated trends in pH (von Brömssen et al., 2023a). This approach identified negative trends in the south-west of Sweden, as well as Mid-Sweden and positive trends in the south-east (Fig. 6, left).

The results of the TWR that was calculated using PCA coordinates (Fig. 6, mid) and the one using PLS coordinates (Fig. 6, right) were plotted in the same geographical system and showed overall similar patterns to the traditional GWR. Negative trends were prevailing in the southwest of Sweden for all models, but the slopes from the TWR-PLS indicated stronger trends than the other two models. In general, the TWR-PLS showed a higher diversity of strong positive and negative trends throughout the country. Although many of the stations indicated in dark red could be classified as outliers because there were very few catchments with similar characteristics, when these were excluded, there was still an indication of generally increasing pH levels in the alpine region in the north of Sweden. GWR suggested a slight increase in pH in the southeast of Sweden which was not reproduced by the TWR models to the same extent. In large parts of the country observed trends were weak (green, yellow, and orange colors).

## Discussion

An evaluation of environmental trends when data is sparse over time for individual stations must rely on smoothing or averaging over several similar stations. Similarity can be expressed by geographical location using geographically weighted regression models (von Brömssen et al., 2023a). However, even geographically close stations can be fundamentally different due to varying catchment characteristics. In this study, we evaluated if similarities can be determined by other station characteristics aside from spatial proximity and if these characteristics can be quantified by coordinate systems based on either PCA or PLS. Subsequently, we suggested that smoothed temporal trends can be computed on these coordinate systems using the same principles as for geographically weighted regression (Brunsdon et al., 1998). To highlight the difference in coordinate systems, we denoted these models as thematically weighted regression.

### Thematically versus geographically weighted trend analyses

Geographically weighted regression, i.e., a grouping by geography, is motivated if important drivers are expected to have regional-scale patterns. For lake water pH levels, such drivers could be recovery from acid deposition or effects of climate change. However, lakes in Sweden are highly diverse due to local variability that could be caused by catchment size, lake location (agriculture, mire, forest), or variations in soil type. Therefore, relying solely on geography could lead to oversimplification and may smooth out prevailing local trends that are only present for a subgroup of stations.

When defining the basis for coordinate systems for thematically weighted regression models, we used variables that describe the initial state of and changes in chemical and climate variables, as well as constant values describing land use in the catchment, location, elevation, and catchment size. While geography is not explicitly included in the model, this information is provided by the temperature gradient, and by elevation, which is highest in the north-west of Sweden. We demonstrate that thematically weighted pH trends were identified as stronger than the geographically weighted pH trends. Naturally, they are also less smoothed out in space and allow for a more in-depth interpretation of the influences of prevailing changes.

### PCA- vs PLS-based coordinate systems

Two different methods have been applied to create the coordinate systems that the thematically weighted regression models were based on. PCA determines the orthogonal factors that best describe the variation in the explanatory variables without taking any response variable into account. Contrastingly, PLS selects the factors that maximize the covariance to a specified response variable. As a proxy for the prevailing trend in pH, we used estimated changes per year based on the two available measurements per station.

Both procedures have clear advantages and disadvantages. PCA creates a coordinate system that is not based on assumptions on prevailing pH trends and, thus, provides objective coordinates that describe the selected explanatory variables. PLS creates an intrinsically more relevant coordinate system, but these could easily be affected by a small number of stations that exhibit large differences in the two registered observations, whereof one could be faulty. In our application, both methods led to reasonable estimates. The removal of stations with suspected outlying values in the PLS factors led to no major changes in the results.

In both PCA and PLS, one of the first factors was based on changes in concentrations describing ionic strength, which in both cases was identified as the most important driver in pH trends. While these variables were relatively clustered in the PCA approach, they were more spread out in the PLS results. There, positive correlations to changes in pH were observed particularly regarding changes in Ca concentrations. This may reflect that Ca is more related to weathering processes generating alkalinity than Na and Cl that correlate more to deposition of neutral sea salt. The correlation between decreases in Ca and pH can also be caused by changes in liming activities, that aim to mitigate acidification and have decreased by 30% between 2008 and 2020 (data from the national liming database) affecting 7% of the survey lakes.

### Selection of drivers

The selection of potential driver variables is, of course, central in this approach, not least when PCA is used. We chose easily available drivers to investigate what can be accomplished with the suggested methods without exerting a large effort of collecting more specific explanatory variables. As a result, much of the variation in trends remains unexplained, with local *R*^2^ values up to approx. 28% in thematically weighted regression based on the PCA approach and 58% for the PLS approach. To establish more informative coordinate systems, variables describing changes in land use or land management for individual catchments could be used. This could include changes in forested areas within catchments, e.g., documented levels of clear-cutting or when the main land-use type in the catchment changes from agricultural to forested land. Both recent and past changes can be of importance as, e.g., afforestation could lead to acidification of soils for a longer time period (McGivney et al., 2019). Similarly, a better description of the lake surroundings could include explicit information of soil and bedrock types as well as soil chemistry. For this, the characteristics of the riparian zone should also be described, rather than just the catchment area. The size of the lake as well as the turnover time can influence how fast temporal trends are observed and should also be included when possible.

In the current approach, we also included initial levels of both pH and other chemicals as well as climatic variables as potential drivers. The period used to quantify these initial levels for chemical variables was 2008 to 2013, while the period for the trend analysis was 2012 to 2023, i.e., the periods overlapped for 2 years. Since observations were made only once every 6 years, this was the only method to define initial values. However, for trends in pH these initial values showed little to no effect on the prevailing trends and in future studies with additional years of monitoring, this problem will decrease.

### Alternative models

A more straightforward approach to include drivers into GWRs would involve explanatory variables directly into the model function. This could be achieved with either basic GWRs, where the smoothing bandwidth is the same for all included variables or, more attractively, using multiscale GWRs (Fotheringham et al., 2017), where separate smoothing bandwidths are determined for each variable, or multiscale geographically and temporally weighted regression models (Li et al., 2025; Wu et al., 2019) that estimate spatial–temporal dynamics within the model. Interpreting bandwidths obtained from these models would also allow us to determine which drivers work on a local, regional, or global scale. However, such models are extremely computationally expensive, making it essential to keep the number of potential driving variables very low.

Using a GWR or TWR approach is strongly motivated by the sparsity of data in the current data set. Since only a small number of observations are made at each station, we have to rely on smoothing over similar stations to obtain reasonably stable results that can be generalized. Without this demand, several other types of statistical models could be investigated. For example, using the observed average change in pH levels as a response to different types of regression models may be possible. Multiple linear regression is likely not an option due to the underlying assumption that all relationships between drivers and the response should be approximately linear. While we use such relationships in our current approaches when defining the PCA and PLS coordinates, this assumption is later relaxed when TWR models are fitted, as these allow the magnitude or sign of trends to change non-parametrically.

Generalized additive models (GAM, Hastie & Tibshirani, 1986) or random forest models (Breiman, 2001) could be used when the linearity assumption is not desired and sparsity of observations is not an issue. Information regarding the geographical location could be included in these models using the geographical coordinates or by using proxies such as temperature gradients or elevation. Spatial dependence of observations should then be directly included into the model. For GAM, this is straightforward using a spatial process on the error term. However, for GAMs, it is also recommended to keep the number of potential variables low, even if shrinkage methods could be used for variable selection (Marra & Wood, 2012). For random forests, adjustments that include spatial aspects into the models have seen increased research efforts (Saha et al., 2023; Talebi et al., 2022). One major difference between GAM and random forest models and TWR is that the former assume that the relationship between temporal changes in the response and the included variables is constant for the entire data set, while TWR lets this relation change by smoothing over the selected coordinates, providing an even more data-driven approach. Within the GWR research, geographical random forests (GRF; Georganos et al., 2021) have been proposed to overcome this limitation. Additional research is needed to identify which model approach is most favorable for given circumstances.

## Conclusion

Exploring changes in both space and time is a demanding but important task in environmental research and water management. Using time series with sampling frequencies high enough to allow for a single site trend analysis often leads to spatial coverage that is too sparse to connect observed changes to specific spatially changing drivers. Additionally, it is often not possible or even desirable to randomly select stations for monitoring of temporal change. A general SLS provides a unique possibility for an unbiased description of the state of the lakes in one country and, thus, covers a wide range of different states of potential drivers. In this study, we have demonstrated that this rather unique type of data in combination with dimension reduction and weighted regression techniques can provide a deeper understanding of what drives changes in pH, especially when a PLS approach is used that allows a proxy of the time trend to be included when the coordinate-basis is established.

## Supplementary Information

Below is the link to the electronic supplementary material.

Supplementary Material 1 (PDF 400 KB)

## Data Availability

All code and selected data is available at https://github.com/claudiavonbromssen/TWR-for-identification-of-driversand in von Brömssen et al. (2025). The remaining data containing climate and land-use is available from the author upon request.
