# Supplementary material for: Thematically weighted regression models for identification of important drivers of environmental trends in lake survey data
Source: Environ Monit Assess. 2025 Oct 16;197(11):1212. doi: 10.1007/s10661-025-14611-4 (PMC12532681; doi:10.1007/s10661-025-14611-4)
Supplement: Supplementary file 1 — Supplementary Material 1 (PDF 400 KB) [file 10661_2025_14611_MOESM1_ESM.pdf]

Supplementary material

Table S.1. Explanatory variables (drivers) included in the analysis.

Variable	Transformation	Type of variable: constant, initial levels, change	Comment
Alk/Acid (mekv/l)	log10	initial	
Ca (mg/l)	log10	initial, change	
Cl (mg/l)	log10	initial, change	
Kond_25 (mS/m)	log10	initial, change	
Mg (mg/l)	log10	initial, change	
Na (mg/l)	log10	initial, change	
pH		initial	
SO_4 (mg/l S)	log10	initial, change	
Air temperature		Long-term mean 1980-2020<br>Initial: Mean 2008-2013,<br>Change: theil slope 2012-2020	
Precipitation		Long-term mean 1980-2020<br>Initial: Mean 2008-2013,<br>Change: theil slope 2012-2020	
Catchment area size		constant	
Altitude		constant	
Mixed forest	centered log ratio	constant	
Mixed coniferous	centered log ratio	constant	
Marine water			Not used
Vegetated_other	centered log ratio	constant	Swedish: hedmark
Rock outcrop	centered log ratio	constant	Swedish: hällmark
Artificial surfaces	centered log ratio	constant	
Other	centered log ratio	constant	Swedish: Ingen täckning
Deciduous forest	centered log ratio	constant	
Semi urban	centered log ratio	constant	
Inland water	centered log ratio	constant	
Forest on wetland	centered log ratio	constant	
Pine forest	centered log ratio	constant	
Young forest	centered log ratio	constant	
Arable land	centered log ratio	constant	
Open wetland	centered log ratio	constant	

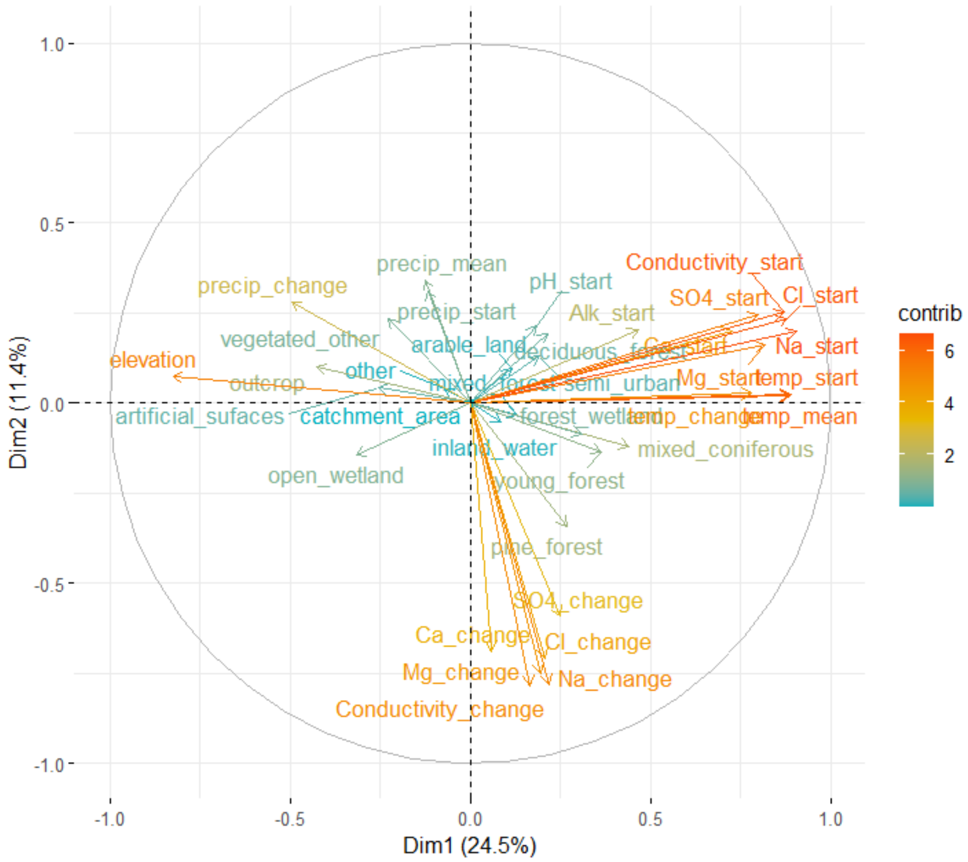

Figure S.1 The first and second principal components defined for the explanatory variables by PCA. The color of the arrows indicates how much they contribute to the two components, with red colors and long arrows indicating strong contributions and short and blue arrows indicating no or little contribution. All variables are presented.

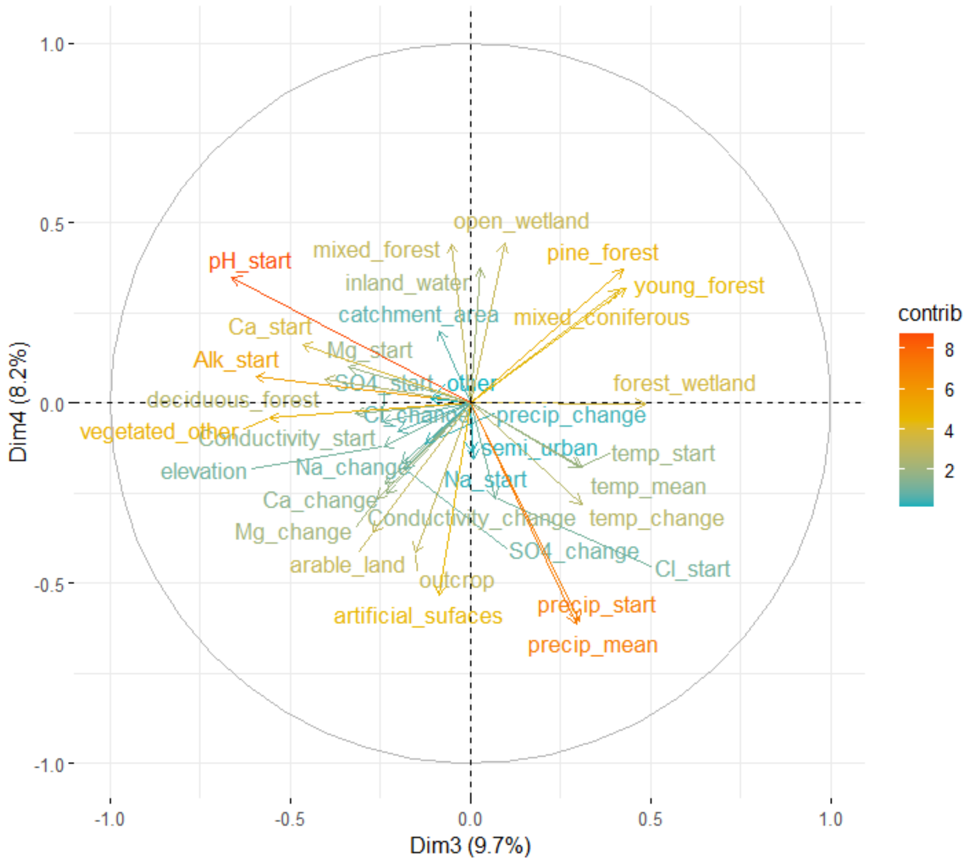

Figure S.2: The third and fourth principal components defined for the explanatory variables by PCA. The color of the arrows indicates how much they contribute to the two components, with red colors and long arrows indicating strong contributions and short and blue arrows indicating no or little contribution.

Table S.2. PCA loadings. Loadings larger than 0.4 and lower than – 0.4 in bold.

**Variable**	**Dim.1**	**Dim.2**	**Dim.3**	**Dim.4**
Ca_start	**0.726**	0.202	**-0.466**	0.163
Cl_start	**0.880**	0.236	0.070	-0.264
Conductivity_start	**0.872**	0.255	-0.319	-0.027
Mg_start	**0.818**	0.162	-0.338	0.100
Na_start	**0.904**	0.199	0.010	-0.157
Alk_start	**0.465**	0.203	**-0.597**	0.074
pH_start	0.184	0.215	**-0.663**	0.35
SO4_start	**0.799**	0.247	-0.243	-0.051
Ca_change	0.059	**-0.688**	-0.236	-0.225
Cl_change	0.205	**-0.707**	-0.202	-0.077
Conductivity_change	0.166	**-0.783**	-0.232	-0.252
Mg_change	0.194	**-0.750**	-0.259	-0.265
Na_change	0.220	**-0.781**	-0.192	-0.167

SO4_change	0.250	**-0.592**	-0.184	-0.182
mixed_forest	0.130	-0.033	-0.052	**0.441**
mixed_coniferous	**0.441**	-0.122	**0.412**	0.316
vegetated_other	-0.229	0.234	-0.558	-0.041
artificial_sufaces	-0.256	0.042	-0.089	-0.535
other	-0.073	0.039	-0.109	0.013
deciduous_forest	0.214	0.194	**-0.405**	0.067
semi_urban	0.191	0.130	0.003	-0.136
inland_water	0.085	-0.052	0.028	0.375
forest_wetland	0.311	-0.086	**0.485**	-0.002
pine_forest	0.267	-0.342	**0.424**	0.371
young_forest	0.364	-0.136	**0.431**	0.320
arable_land	0.115	0.097	-0.269	-0.359
open_wetland	-0.318	-0.145	0.094	**0.444**
outcrop	**-0.428**	0.100	-0.153	**-0.416**
temp_mean	**0.886**	0.019	0.31	-0.179
precip_mean	-0.126	0.341	0.294	**-0.614**
temp_start	**0.888**	0.023	0.304	-0.178
precip_start	-0.119	0.310	0.302	-0.606
temp_change	**0.781**	0.027	0.308	-0.279
precip_change	**-0.496**	0.280	-0.125	-0.112
catchment_area	0.008	0.008	-0.086	0.199
elevation	**-0.826**	0.074	-0.241	-0.121

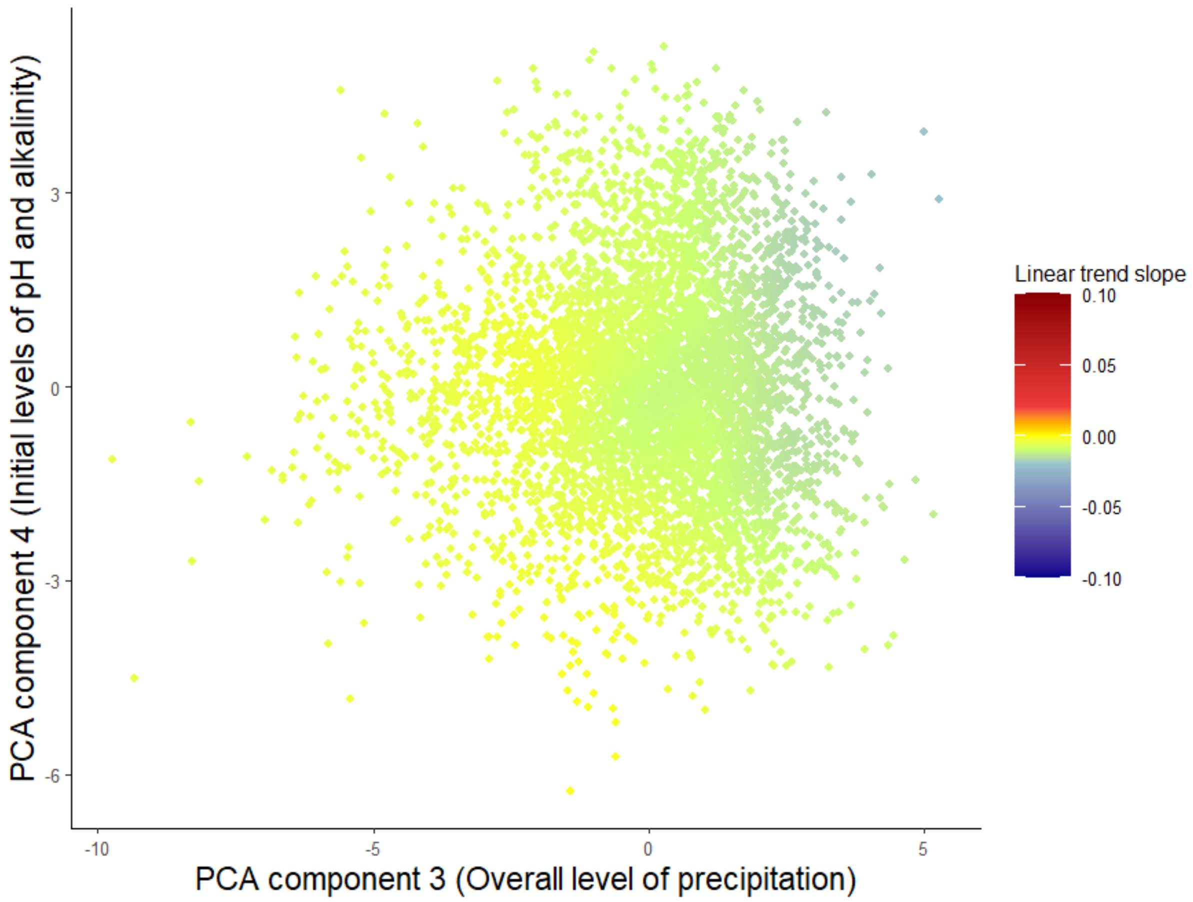

Figure S.3: The results of the TWR for trends in pH 2012-2023 on the third and fourth PCA coordinates. The x-axis represents the third principal component and the y-axis the fourth principal component equivalent to the components presented in Figure S.2.

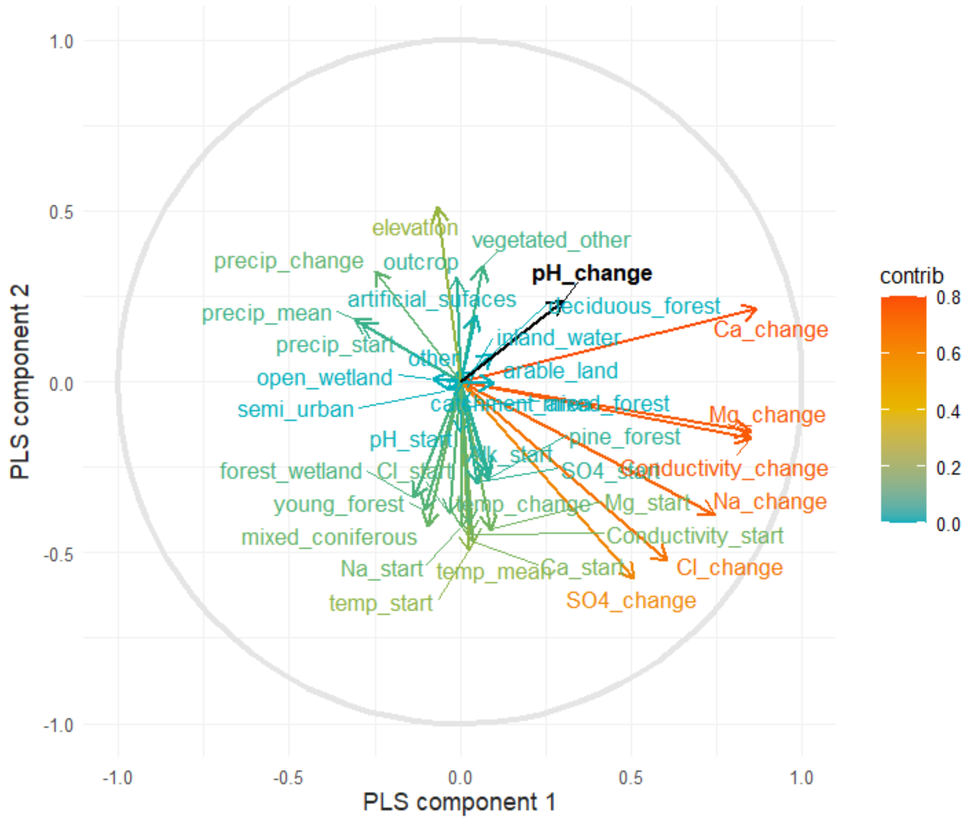

Figure S.4: The first two components defined in the PLS. The color and length of the arrows indicate the extent to which different variables contribute to explaining the variation in the two components, using R^2 values as an index. All variables are presented.

Table S.3. PLS loadings. Loadings larger than 0.4 and lower than – 0.4 in bold.

Variable	PLS 1	PLS 2	PLS 3	PLS 4
Ca_start	0.023	-0.382	0.218	-0.215
Cl_start	-0.017	-0.315	0.334	-0.278
Conductivity_start	0.018	-0.363	0.301	-0.277
Mg_start	0.053	-0.352	0.280	-0.193
Na_start	0.002	-0.347	0.336	-0.243
Alk_start	0.049	-0.217	0.131	-0.247
pH_start	0.002	-0.123	0.024	-0.212
SO4_start	0.029	-0.241	0.311	-0.263
Ca_change	**0.521**	0.173	0.047	0.109
Cl_change	0.363	**-0.426**	-0.097	0.041
Conductivity_change	**0.509**	-0.133	-0.023	0.011
Mg_change	**0.510**	-0.119	-0.008	-0.051
Na_change	**0.446**	-0.317	-0.054	0.065
SO4_change	0.306	**-0.467**	-0.097	-0.117
mixed_forest	0.006	-0.059	0.061	0.122

mixed_coniferous	-0.058	-0.343	0.149	0.305
vegetated_other	0.038	0.274	-0.065	-0.391
artificial_sufaces	0.027	0.157	-0.102	-0.168
other	-0.007	0.017	-0.041	-0.068
deciduous_forest	0.057	0.067	0.099	-0.37
semi_urban	-0.024	-0.021	0.087	-0.123
inland_water	0.024	0.024	0.066	0.09
forest_wetland	-0.080	-0.276	0.101	0.264
pine_forest	0.048	-0.231	0.101	**0.474**
young_forest	-0.063	-0.306	0.126	0.348
arable_land	0.059	-0.004	0.025	-0.256
open_wetland	-0.048	0.007	-0.137	0.274
outcrop	-0.007	0.248	-0.16	-0.203
temp_mean	0.016	**-0.403**	0.321	-0.085
precip_mean	-0.185	0.148	-0.01	-0.217
temp_start	0.016	-0.403	0.322	-0.089
temp_change	0.016	-0.339	0.285	-0.119
precip_start	-0.172	0.137	-0.009	-0.191
precip_change	-0.150	0.263	-0.167	-0.145
catchment_area	0.005	0.004	0.004	-0.019
elevation	-0.040	**0.415**	-0.293	-0.042
